# Cholesterol 7 alpha-hydroxylase (*CYP7A1*) gene polymorphisms are associated with increased LDL-cholesterol levels and the incidence of subclinical atherosclerosis

**DOI:** 10.17305/bb.2024.10764

**Published:** 2024-08-28

**Authors:** Gilberto Vargas-Alarcón, Rosalinda Posadas-Sánchez, Oscar Pérez-Méndez, José Manuel Fragoso

**Affiliations:** 1 Direccion de Investigación, Instituto Nacional de Cardiología Ignacio Chávez, Mexico City, México; 2 Departmento de Endocrinología, Instituto Nacional de Cardiología Ignacio Chávez, Mexico City, México; 3 Departmento de Biología Molecular, Instituto Nacional de Cardiología Ignacio Chávez, Mexico City, México

**Keywords:** Genetics, susceptibility, subclinical atherosclerosis (SA), cholesterol 7 alpha-hydroxylase (CYP7A1)

## Abstract

The cholesterol 7 alpha-hydroxylase (CYP7A1) enzyme plays an important role in the conversion of cholesterol to bile acid, contributing to the reduction of cholesterol plasma levels in normal conditions. Nonetheless, recent studies have shown that some genetic variants in the enhancer and promoter regions of the *CYP7A1* gene reduce the expression of the CYP7A1 enzyme, increasing plasma lipid levels, as well as the risk of developing coronary heart disease. The aim of this work was to explore whether the genetic variants (rs2081687, rs9297994, rs10107182, rs10504255, rs1457043, rs8192870, and rs3808607) of the *CYP7A1* gene are involved in subclinical atherosclerosis (SA) and plasma lipid levels. We included 416 patients with SA with coronary artery calcium (CAC) greater than zero, and 1046 controls with CAC ═ 0. According to the inheritance models (co-dominant, dominant, recessive, over-dominant, and additive), the homozygosity of the minor allele frequencies of seven analyzed polymorphisms showed a high incidence of SA (*P* < 0.05). In a subanalysis performed including only the patients with SA, the same single nucleotide polymorphisms (SNPs) were associated with increased low-density lipoprotein cholesterol (LDL-C) levels. On the other hand, our findings showed that the haplotype (*TGCGCTG*) increases the risk of developing SA (*P* < 0.05). In conclusion, the rs2081687, rs9297994, rs10107182, rs10504255, rs1457043, rs8192870, and rs3808607 polymorphisms of *CYP7A1* confer a risk of developing SA and elevated LDL-C levels. Our results suggest that the CYP7A1 is involved in the incidence of SA through the increase in the plasma lipid profile.

## Introduction

Dyslipidemia is characterized by an increase in plasma lipid profile concentration, being one of the most important risk factors in the development of atherosclerosis (AS), including subclinical atherosclerosis (SA) [1–3]. SA is an atheromatous disease in which there are no signs, symptoms, or events associated with AS [4, 5]. SA may be diagnosed by a coronary artery calcification (CAC) score greater than zero. Furthermore, this CAC score provides a measure of the extent of the atherosclerotic lesion, being considered a diagnostic marker for SA [4–8]. Cholesterol 7 alpha-hydroxylase (CYP7A1) is an enzyme involved in the biochemical synthesis of bile acids from cholesterol, representing one of the main cholesterol elimination pathways in the hepatocyte [9–11]. Accordingly, experimental studies have shown that low expression of CYP7A1 diminishes the catabolism of cholesterol, thus increasing low-density lipoprotein -cholesterol (LDL-C) plasma levels [9, 10, 12–17]. The increased LDL-C plasma levels would then favor the development of AS [2, 3]. Cholesterol 7 alpha-hydroxylase is encoded by the *CYP7A1* gene located on chromosome 8, regions q11-12 [10]. Additionally, recent reports have shown that some single nucleotide polymorphisms (SNPs) located in the promoter and enhancer regions (rs2081687 *C/T,* rs9297994 *G/A*, rs10107182 *C/T,* rs10504255 *A/G* rs1457043 *C/T,* rs8192870 *G/T*, and rs3808607 *G/T*) are associated with *CYP7A1* mRNA expression, as well as with hypercholesterolemia, coronary heart disease, and type 2 diabetes mellitus (DM2) [9, 10, 12–17].

Therefore, considering the central role of CYP7A1 activity in cholesterol catabolism, in this study, we hypothesize that CYP7A1 gene polymorphisms above-mentioned are associated with elevated LDL-C plasma levels, and a consequent higher risk of SA. The objective of this study was to look for the potential statistical association of the rs2081687 *C/T,* rs9297994 *G/A*, rs10107182 *C/T,* rs10504255 *A/G* rs1457043 *C/T,* rs8192870 *G/T*, and rs3808607 *G/T* SNPs with the risk of developing SA, and with the plasma lipid profile, particularly LDL-C and total cholesterol.

## Materials and methods

### Characteristics of the study population

This cross-sectional study is nested in the Genetics of Atherosclerotic Disease (GEA) study, which investigates the associations of gene polymorphisms with AS in Mexican individuals [18]. The GEA cohort was recruited from June 2008 to January 2013 at the Instituto Nacional de Cardiología. The 1462 Mexican mestizo volunteers included in the present study were enrolled in the GEA cohort after a medical examination and health questionnaire. The main inclusion criteria for these 1462 individuals were the absence of a personal or familial history of coronary heart disease or current or previous congestive heart failure. Exclusion criteria were liver, renal, thyroid, and oncological diseases, determined by clinical chemistry and medical exploration [18]. The 1462 volunteers in this study were born in Mexico and considered Mexican Mestizo based on autochthonous and Caucasian and/or Black origin. Once included in the cohort, the 1462 volunteers in this study underwent a computed tomography scan to assess the CAC score [19]. A CAC score > 0 without clinical symptoms of coronary artery disease was established as a diagnosis of SA.

### Clinical and laboratory measurements

Cholesterol and triglyceride plasma levels were performed in plasma samples and were determined using commercially available kits (Randox Laboratories, Crumlin, UK). High-density lipoprotein-cholesterol (HDL-C) was determined after selective precipitation of apolipoprotein B-containing lipoproteins with phosphotungstic acid-Mg^2+^. The Friedewald formula was used to estimate the LDL-C plasma concentration [20] if triglyceride concentrations were < 400 mg/dL. Patients were considered to have diabetes when their fasting glucose levels were ≥ 126 mg/dL, they had a previous diagnosis of the disease, or were using antidiabetic medications (https://www.msdmanuals.com/professional/endocrine-and-metabolic-disorders/diabetes-mellitus-and-disorders-of-carbohydrate-metabolism/diabetes-mellitus-dm#v29299021). Subjects were considered hypertensive when they had systolic blood pressure values ≥ 130 mmHg, and/or diastolic blood pressure ≥ 90 mmHg, or were using anti-hypertensive drugs when blood samples were drawn for this study, according to the MSD manual (https://www.msdmanuals.com/professional/cardiovasculardisorders/hypertension/hypertension?query=hypertension (accessed on Dec 2, 2023)).

### Genetic analysis

DNA samples were obtained from whole blood as previously described [21]. *CYP7A1* gene polymorphisms (rs2081687 *C/T,* rs9297994 *G/A*, rs10107182 *C/T,* rs10504255 *A/G* rs1457043 *C/T,* rs8192870 *G/T*, and rs3808607 *G/T*) were analyzed in patients with SA and control individuals using TaqMan assays on a QuantStudio 12K Flex Real-Time PCR system from Applied Biosystems, Foster City, USA. In addition, information regarding these polymorphisms, such as chromosome position, base change, and gene location, is shown in Table S1.

### Ethical statement

This work complies with the statements of the Declaration of Helsinki and was approved by the local Ethics Committee under project number 23-1361. All participants enrolled in this study signed the corresponding informed consent.

### Statistical analysis

Data distribution was determined by the Shapiro–Francia test. Variables in the SA and control groups were compared using Student’s *t*-test and represented as mean ± SD, or by Mann–Whitney *U* non-parametric tests, represented as the median and interquartile interval [25th–75th], when the variable distribution was normal or non-normal, respectively. For categorical variables, Fisher’s exact test or chi-squared test was performed. The association of *CYP7A1* gene polymorphisms with SA was analyzed using additive, codominant, dominant, over-dominant (heterozygous), and recessive models of inheritance [22, 23]. These analyses were performed using logistic regression. Logistic regression models included age, gender, blood pressure, and diabetes incidence as confounding variables. The *P* values (*P*) were corrected using the Bonferroni method, corresponding to the number of tests for each SNP based on the five inheritance models. The results of the analyses were presented as odds ratios (OR) with 95% confidence intervals. The power of the statistical analyses was set to 0.80 (OpenEpi available online, http://www.openepi.com/SampleSize/SSCC.htm). *P* < 0.05 was fixed for statistical significance.

Haplotypes and linkage disequilibrium (LD) were analyzed with Haploview version 4.1 (Broad Institute of Massachusetts Institute of Technology and Harvard University, Cambridge, MA, USA). Haploview uses the international HapMap project database and the standard EM algorithm to estimate the phase of haplotypes, considering the combination of analyzed alleles in one or more genes that can segregate together due to the closeness among them. This analysis estimates the maximum-likelihood values to obtain the D’ logarithm of the odds (LOD), and *r*^2^ [24].

### Analysis of the association between CYP7A1 genotypes with cardiovascular risk factors

To explore the potential contribution of *CYP7A1* gene polymorphisms to triglycerides, total cholesterol, HDL-C, LDL-C, glucose, BMI, and systolic and diastolic blood pressures, individuals were grouped based on their genotype for each SNP. Comparisons among carriers of different genotypes were performed by ANOVA after the logarithmic transformation of non-normally distributed variables. Variance homogeneity was evaluated using the Levine test and confirmed by the *F* test.

## Results

### Characteristics of the study subjects

On the basis of the CAC score [19], 416 individuals were classified as patients with SA, meaning that they had a CAC score > 0, and 1046 were assigned to the control group (CAC score ═ 0). Table 1 shows the biochemical and anthropometric characteristics of controls and SA patients. With the exception of body mass index, total cholesterol, triglycerides and smoking habits, the risk variables such as glucose, HDL-C, and LDL-C levels, as well as the incidence of hypertension, and DM2, were higher in patients with SA than in control individuals.

**Table 1:** Anthropometric and clinical characteristics of the patients with SA and control individuals

**Characteristics**	**SA patients (*n* ═ 416)**	**Controls (*n* ═ 1046)**	***P***
Age (years)	58.3 ± 8.74	51.3 ± 8.9	<0.001
BMI (kg/m^2^)	28.5 ± 3.9	28.2 ± 8.9	0.507
Gender *n* (%), Male	310 (74.5)	425 (40.6)	<0.001
Female	106 (25.4)	621 (59.3)	<0.001
Hypertension, Yes, *n* (%)	155 (37.2)	200 (19.1)	<0.001
Type 2 diabetes mellitus, Yes, *n* (%)	91 (21.8)	109 (10.4)	<0.001
Smoking, *n* (%)	97 (23.3)	232 (22.1)	0.638
Blood pressure (mmHg), Systolic	121.5 [111–133]	112 [103–122]	<0.001
Blood pressure (mmHg), Diastolic	74 [68–81]	70 [65–76]	<0.001
Glucose (mg/dL)	94 [86–108]	90 [84–97]	<0.001
Total cholesterol (mg/dL)	196 [168–219]	190 [167–211]	0.070
HDL-C (mg/dL)	43 [36–50]	45 [36–55]	0.015
LDL-C (mg/dL)	123 [101–145]	116 [96–134]	0.014
Triglycerides (mg/dL)	154 [118–204]	145 [107–202]	0.063

Data were collected at recruitment. Gender, hypertension, type 2 diabetes mellitus, and smoking are expressed as *n* (frequency), and *P* values was calculated to chi-square. Other variables are expressed as Mean ± SD or median [25th–75th interquartile interval] for normally or non-normally distributed variables and groups were compared by *t*-test or Mann–Whitney *U* test, respectively. HDL-C: High-density lipoprotein-cholesterol; LDL-C: Low-density lipoprotein cholesterol; BMI: Body mass index.

### Association of CYP7A1 polymorphisms with SA

The genotypic frequencies of CYP7A1 gene polymorphisms in SA patients and controls were in Hardy–Weinberg equilibrium (*P* > 0.05). The allelic and genotypic distribution of the seven polymorphisms considered in this study were different in SA patients compared to controls (*P* < 0.05) (Table S2).

The analysis of polymorphisms with the incidence of SA, according to the inheritance models, is shown in Table 2. The analysis of the rs2081687 *C/T* SNP showed that the carrier individuals of one or two copies of the *T* allele had an increased risk of developing SA under four models (Table 2). Additionally, the carriers of one or two copies of the rs9297994 *G* allele of the *G/A* SNP showed a higher risk of developing SA (Table 2). Similar findings were observed with the rs10107182 *C/T* SNP; this analysis showed that the carriers of one or two copies of the *C* allele had an increased risk of developing SA. Moreover, individuals who presented one or two copies of the rs10504255 *G* allele had the highest risk of developing SA, under four of the inheritance models (Table 2). Carrier individuals of one or two copies of the rs1457043 SNP *C* allele were associated with the highest risk of developing SA. In addition, the rs8192870 *G/T* polymorphism analysis showed that the carriers of one or two copies of the *T* allele also had an increased risk of developing SA. Finally, the analysis of the rs3800867 *G/T* SNP showed that the individuals who presented one or two copies of the *G* allele had an increased risk of developing SA under codominant, recessive, and additive models (Table 2).

**Table 2:** Association of the *CYP7A1* polymorphisms with SA accordance to the inheritance models

SNP (rsID-number)/*Inehritance models	**Genotype**	**SA patients *n* ═ 416 (*n*(%))**	**Controls *n* ═ 1046 (*n*(%))**	**OR (95% CI)**	**pC**
rs2081687 *T/C*					
Co-dominant	*CC CT TT*	262 (0.629) 137 (0.329) 17 (0.041)	759 (0.726) 269 (0.257) 18 (0.017)	1.46 (1.07–1.95)	0.012
Dominant	*CC CT + TT*	262 (0.629) 154 (0.370)	759 (0.726) 287 (0.274)	1.50 (1.11–1.99)	0.006
Recessive	*CC + CT TT*	399 (0.959) 17 (0.041)	1028 (0.983) 18 (0.017)	1.81 (0.80–3.99)	0.151
Over-dominant	*CC + TT CT*	279 (0.671) 137 (0.329)	777 (0.743) 269 (0.257)	1.41 (1.04–1.89)	0.017
Additive	*–*	–	–	1.45 (1.11–1.85)	0.004
rs9297994 *G/A*					
Co-dominant	*AA AG GG*	266 (0.639) 135 (0.325) 15 (0.036)	767 (0.733) 264 (0.252) 15 (0.014)	1.49 (1.12–2.00)	0.014
Dominant	*AA AG + GG*	266 (0.639) 150 (0.361)	767 (0.733) 279 (0.267)	1.53 (1.13–2.00)	0.005
Recessive	*AA + AG GG*	401 (0.964) 15 (0.036)	1031 (0.986) 15 (0.014)	1.87 (0.74–4.54)	0.177
Over-dominant	*AA + GG AG*	281 (0.674) 135 (0.325)	782 (0.748) 264 (0.252)	1.46 (1.07–1.95)	0.012
Additive	*–*	–	–	1.48 (1.13–1.92)	0.004
rs1010718 2 *C/T*					
Co-dominant	*TT TC CC*	264 (0.635) 137 (0.329) 15 (0.036)	765 (0.731) 266 (0.254) 15 (0.014)	1.51 (1.13–2.00)	0.005
Dominant	*TT TC + CC*	264 (0.635) 152 (0.365)	765 (0.731) 281 (0.269)	1.55 (1.15–2.00)	0.003
Recessive	*TT + TC CC*	401 (0.964) 15 (0.036)	1031 (0.986) 15 (0.014)	1.87 (0.72–4.54)	0.179
Over-dominant	*TT + CC TC*	279 (0.671) 137 (0.329)	780 (0.746) 266 (0.254)	1.47 (1.08–1.97)	0.007
Additive	*–*	–	–	1.50 (1.11–1.93)	0.001
rs10504255 *G/A*					
Co-dominant	*AA AG GG*	265 (0.637) 135 (0.325) 16 (0.038)	774 (0.740) 255 (0.244) 17 (0.016)	1.54 (1.13–1.99)	0.008
Dominant	*AA AG + GG*	265 (0.637) 151 (0.363)	774 (0.740) 272 (0.259)	1.58 (1.16–2.00)	0.001
Recessive	*AA + AG GG*	400 (0.962) 16 (0.038)	1029 (0.984) 17 (0.016)	1.88 (0.79–4.36)	0.149
Over-dominant	*AA + GG AG*	281 (0.675) 135 (0.325)	791 (0.756) 255 (0.244)	1.49 (1.10–2.00)	0.006
Additive	–	–	–	1.52 (1.16–1.95)	0.001
rs1457043 *C/T*					
Co-dominant	*TT CT CC*	207 (0.498) 169 (0.406) 40 (0.096)	582 (0.556) 403 (0.385) 61 (0.058)	1.79 (1.13–2.74)	0.028
Dominant	*TT CT + CC*	207 (0.498) 209 (0.502)	582 (0.556) 464 (0.444)	1.27 (1.04–1.57)	0.041
Recessive	*TT + CT CC*	376 (0.904) 40 (0.096)	985 (0.942) 61 (0.058)	1.66 (1.03–2.53)	0.021
Over-dominant	*TT + CC CT*	247 (0.594) 169 (0.406)	643 (0.615) 403 (0.385)	1.11 (0.86–1.40)	0.419
Additive	–	–	–	1.27 (1.02–1.52)	0.009
rs81922870 *T/G*					
Co-dominant	*GG GT TT*	258 (0.620) 142 (0.341) 16 (0.038)	737 (0.705) 289 (0.276) 20 (0.019)	1.43 (1.07–1.92)	0.038
Dominant	*GG GT + TT*	258 (0.620) 158 (0.380)	737 (0.705) 309 (0.295)	1.44 (1.04–1.95)	0.011
Recessive	*GG + GT TT*	400 (0.962) 16 (0.038)	1026 (0.981) 20 (0.019)	1.36 (0.60–3.00)	0.473
Over-dominant	*GG + TT GT*	274 (0.659) 142 (0.341)	757 (0.724) 289 (0.276)	1.40 (1.03–1.85)	0.019
Additive	–	–	–	1.37 (1.02–1.75)	0.012
rs3800867 *G/T*					
Co-dominant	*TT GT GG*	211 (0.507) 167 (0.401) 38 (0.091)	590 (0.564) 399 (0.381) 57 (0.055)	1.78 (1.11–2.82)	0.027
Dominant	*TT GT + GG*	211 (0.507) 205 (0.493)	590 (0.564) 456 (0.436)	1.25 (0.95–1.60)	0.077
Recessive	*TT + GT GG*	378 (0.909) 38 (0.091)	989 (0.945) 57 (0.055)	1.66 (1.07–2.53)	0.024
Over-dominant	*TT + GG GT*	249 (0.599) 167 (0.401)	647 (0.619) 399 (0.295)	1.10 (0.86–1.37)	0.473
Additive	–	–	–	1.26 (1.04–1.50)	0.012

*The inheritance models were design based in the minor allele. Co-dominant model that compares the subgroup of homozygous individuals carrying the minor allele with homozygotes of the major allele. Dominant model compares the subgroup of homozygous individuals carrying the major allele with the subgroup conformed by heterozygotes and minor allele homozygotes. Recessive model compares the subgroup conformed by heterozygotes and major allele homozygotes vs homozygotes of the minor allele. Over-dominant model compares the subgroup conformed by homozygotes of the major allele and homozygotes of the minor allele vs heterozygotes. Additive model compares the subgroup major allele carriers with both, heterozygotes and minor allele homozygotes. All models were analyzed by logistic regression including gender, age, hypertension, type 2 diabetes mellitus, and smoking habit as confounding variables. SNP: Single nucleotide polymorphism; SA: Subclinical atherosclerosis; OR: Odds ratio; CI: Confidence interval; *pC:* Corrected *P* value.

### LD analysis

**Figure 1.**
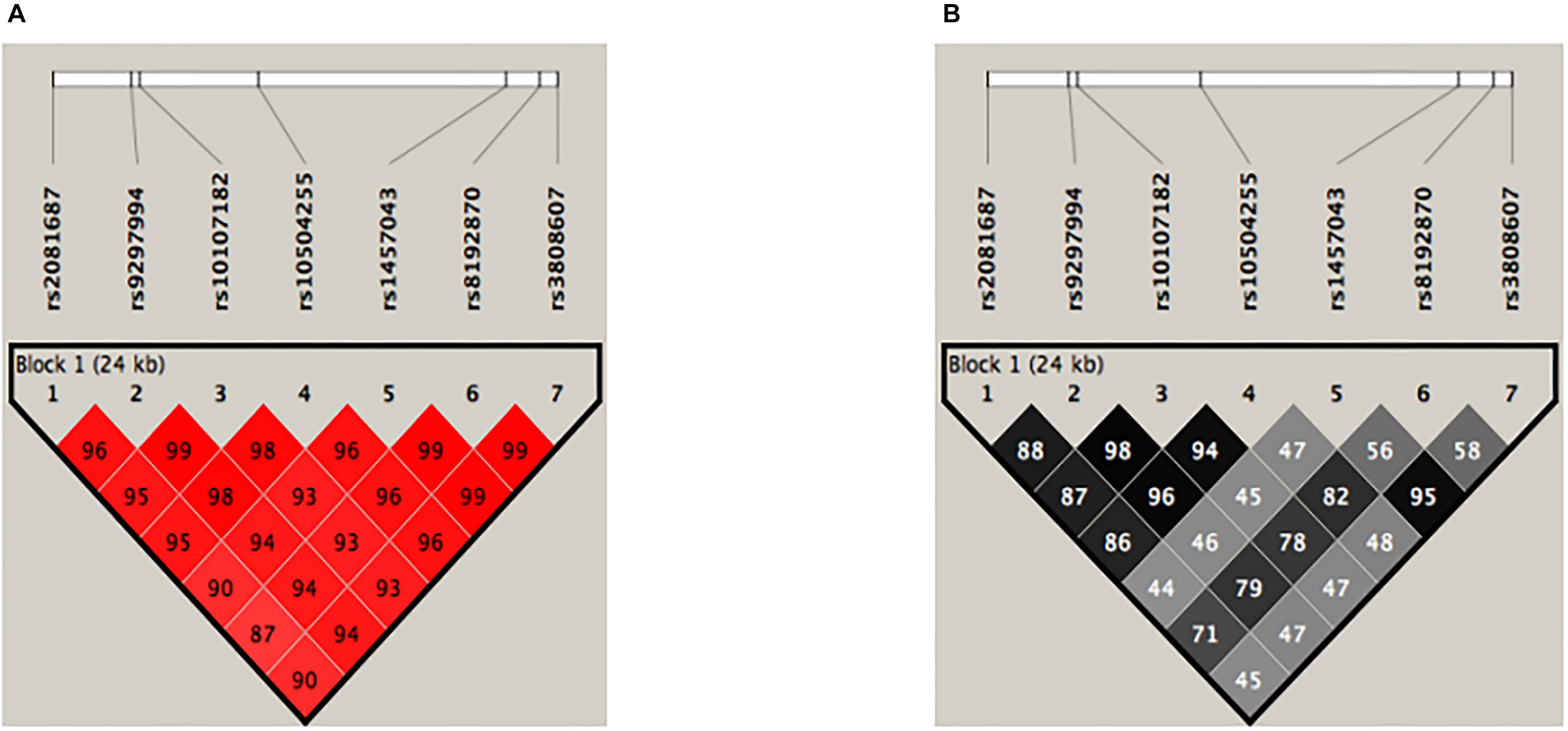
**LD analysis for haplotype analyses.** (A) In red, the block formed by SNPs (rs2081687, rs9297994, rs10107182, rs10504255, rs1457043, rs8192870, and rs3808607) shows a strong LD (D’ > 0.85), increasing the probability that this block may segregate together; (B) In shades of gray, the block formed by the same SNPs showed that rs1457043, rs8192870, and rs3808607 polymorphisms recombine more that rs2081687, rs9297994, rs10107182, and rs10504255 SNPs (*r*^2^ < 0.80). The D’ and *r*^2^ values are presented ×100. Data were analyzed with Haploview, version 4.1 (Broad Institute of Massachusetts Institute of Technology and Harvard University, Cambridge, MA, USA). LD: Linkage disequilibrium.

As shown in Figure 1, the block formed by the seven polymorphisms considered in this study had a strong LD (D’ > 0.85), increasing the probability that these polymorphisms may be inherited together. Nonetheless, the analysis of r^2^ showed that the rs8192870 *G/T*, rs3808607 *G/T,* and rs1457043 *C/T* polymorphisms recombine more than rs10504255 *A/G*, rs9297994 *G/A*, rs2081687 *C/T,* and rs10107182 *C/T*, SNPs (*r*^2^ < 0.80). This analysis revealed two haplotypes. “*CATATGT*” and “*TGCGCTG*” had different frequencies in patients with SA compared to controls (Table 3). The “*CATATGT*” haplotype was associated with protection against the development of SA, while the “*TGCGCTG”* haplotype represented a risk of SA (OR ═ 1.56, *P* ═ 5×10^−5^).

### Effect of the genotypes of CYP7A1 SNPs on plasma lipid levels

Recent data have shown that the cholesterol 7α-hydroxylase is associated with high plasma lipid levels, familial hypercholesterolemia, and cardiovascular diseases [10–17, 25]. In this context, we created subgroups based on the genotype of each one of the studied SNPs, to compare BMI, blood pressure, glucose, as well as the plasma lipid concentrations. The results of these sub-analyses demonstrated that the homozygous carriers of the minor allele of any of the seven polymorphisms analyzed in this study had increased LDL-C levels (*P* < 0.05) (Table 4). Also, rs3808607 *GG* genotype showed lower concentrations of the triglycerides (*P* < 0.05) (Table 4).

**Table 3:** Distribution of haplotypes between *CYP7A1* gene polymorphisms in the study groups

*Polymorphic site	**SA *n* ═ 416**	**Controls *n* ═ 1046**	**OR**	**95% CI**	***P***
Block haplotype	Hf (%)	Hf (%)			
*C A T A T G T*	0.681	0.735	0.76	0.64–0.91	0.003
*T G C G C T G*	0.186	0.128	1.56	1.26–1.94	5×10^4^
*C A T A C G G*	0.081	0.081	1.0	0.74–1.34	0.998
*C A T A C T G*	0.013	0.023	0.57	0.29–1.10	0.093

Haplotypes were analyzed using Haploview software, version 4.1 (Broad Institute of Massachusetts Institute of Technology and Harvard University, Cambridge, MA, USA). *The polymorphisms order, and the allele combination to the haplotypes is according to the position in the chromosome 8q11-12 (rs2081687 *T/C*-rs9297994 *G/A-*rs10107182 *C/T-*rs10504255 *G/A-*rs1457043 *C/T-*rs8192870 *T/G-*rs3808607 *G/T*) as depicted in Figure 1. SA: Subclinical atherosclerosis; Hf: Haplotype frequency; *P*: *P* value; OR: Odds ratio; CI: Confidence interval.

**Table 4:** Plasma lipid concentrations and cardiovascular risk factors in patients with SA grouped by *CYP7A1* genotypes

**Gene/Parameters of SA**	**SNP/Genotypes**			
***CYP7A1***	***rs2081687 T/C***			
	****CC* (*n* ═ *262*)***	****CT* (*n* ═ *137*)***	****TT* (*n* ═ *17*)***	P*
BMI (kg/m^2^)	28 [25.6–31]	28.2 [26–31]	29.7 [27.5–31]	0.501
Systolic blood pressure (mmHg)	121 [111–132]	121 [112–132]	127 [119–140]	0.704
Diastolic blood pressure (mmHg)	74 [68–81]	74 [68–82]	77.5 [73.5–86]	0.547
Glucose (mg/dL)	94 [87–107]	91 [85–102]	98 [90–104]	0.170
Total cholesterol (mg/dL)	193.6 [164–222]	200 [175–217]	191 [187–214]	0.556
HDL-cholesterol (mg/dL)	42.5 [36–49]	45 [38–54]	44 [37–49]	0.096
LDL-cholesterol (mg/dL)	121.4 [99–146]	128 [107–142]	139 [132–148]	0.023
Triglycerides (mg/dL)	159 [118–211]	144 [117–193]	146 [134–181]	0.303
***CYP7A1***	***rs9297994 G/A***			
	***AA* (*n* ═ *266*)**	***AG* (*n* ═ *135*)**	***GG* (*n* ═ *15*)**	P*
BMI (kg/m^2^)	28.6 [26–31]	28.4 [26–31]	29.7 [26–31]	0.853
Systolic blood pressure (mmHg)	121 [111–132]	122 [112–133]	127.5 [111–143]	0.633
Diastolic blood pressure (mmHg)	74 [68–80]	74.5 [69–82]	77.5 [71–83]	0.684
Glucose (mg/dL)	94 [87–107]	91 [85–102]	98 [85–105]	0.081
Total cholesterol (mg/dL)	195 [165–222]	200 [175–216]	201 [188–210]	0.364
HDL-cholesterol (mg/dL)	42.5 [36–49]	45 [37–54]	45 [37.6–52]	0.162
LDL-cholesterol (mg/dL)	121.5 [100–146]	127.8 [106–142]	142 [132–150]	0.027
Triglycerides (mg/dL)	159 [118–211]	144 [116–194]	141.5 [117–173]	0.286
***CYP7A1***	***rs10107182 C/T***			
	***TT* (*n* ═ *264*)**	***TC* (*n* ═ *137*)**	***CC* (*n* ═ *15*)**	P*
BMI (kg/m^2^)	28 [26–31]	28 [26–31]	29.7 [26–31]	0.720
Systolic blood pressure (mmHg)	121 [111–132]	121 [111–132]	127 [111–143]	0.724
Diastolic blood pressure (mmHg)	74 [68–80]	74 [68–81]	77 [71–83]	0.559
Glucose (mg/dL)	94 [87–107]	93.5 [86–104]	98 [85–105]	0.654
Total cholesterol (mg/dL)	195 [165–223]	196 [167–219]	198 [191–207]	0.571
HDL-cholesterol (mg/dL)	42 [36–49]	43 [36–50]	45 [38–52]	0.685
LDL-cholesterol (mg/dL)	121 [99–146]	123 [100–145]	145 [142–148]	0.026
Triglycerides (mg/dL)	159 [118–212]	155 [118–205]	141 [116–173]	0.495
*CYP7A1*	*rs10504255 G/A*			
	***AA* (*n* ═ *265*)**	***AG* (*n* ═ *135*)**	***GG* (*n* ═ *16*)**	P*
BMI (kg/m^2^)	28 [27–31]	28 [26–31]	29 [26–31]	0.844
Systolic blood pressure (mmHg)	121 [110–132]	122 [112–133]	126 [109–138]	0.804
Diastolic blood pressure (mmHg)	74 [68–80]	75 [69–82]	78 [67–83]	0.348
Glucose (mg/dL)	94 [86–107]	93 [86–102]	91 [83–104]	0.386
Total cholesterol (mg/dL)	195 [165–222]	201 [175–217]	204 [201–210]	0.155
HDL-cholesterol (mg/dL)	43 [36–49]	45 [37–54]	46 [38–54]	0.144
LDL-cholesterol (mg/dL)	121 [99.6–146]	128 [106–142]	143 [139–153]	0.015
Triglycerides (mg/dL)	159 [118–211]	150 [118–195]	140 [99–169]	0.178
***CYP7A1***	***rs1457043 C/T***			
	****TT* (*n* ═ *207*)***	****CT* (*n* ═ *169)****	****CC* (*n* ═ *40*)***	P*
BMI (kg/m^2^)	28 [25–31]	28 [26–30]	28 [26–31]	0.321
Systolic blood pressure (mmHg)	122 [111–133]	121 [112–132]	125 [110–138]	0.669
Diastolic blood pressure (mmHg)	75 [69–81]	73 [67–80]	76 [69–83]	0.402
Glucose (mg/dL)	93 [86–108]	94 [86–103]	94 [87–104]	0.761
Total cholesterol (mg/dL)	193 [165–222]	202 [168–218]	203 [183–213]	0.615
HDL-cholesterol (mg/dL)	43 [36–50]	43 [36–49]	47 [40–54]	0.144
LDL-cholesterol (mg/dL)	120 [100–145]	128 [103–145]	139 [131–147]	0.002
Triglycerides (mg/dL)	159 [115–215]	157 [123–200]	137 [99–162]	0.066
***CYP7A1***	***rs8192870 G/T***			
	****GG* (*n* ═ *258*)***	****GT* (*n* ═ *142*)***	****TT* (*n* ═ *16*)***	P*
BMI (kg/m^2^)	28 [26–31]	28 [26–31]	29 [26–31]	0.844
Systolic blood pressure (mmHg)	121 [111–132]	122 [112–113]	130 [111–138]	0.863
Diastolic blood pressure (mmHg)	74 [68–80]	74 [68–81]	80 [68–83]	0.417
Glucose (mg/dL)	94 [87–108]	92 [86–102]	96 [84–108]	0.200
Total cholesterol (mg/dL)	195 [167–222]	202 [175–218]	203 [201–210]	0.498
HDL-cholesterol (mg/dL)	43 [36–50]	44 [37–52]	47 [41–54]	0.107
LDL-cholesterol (mg/dL)	121 [100–146]	132 [112–144]	145 [143–149]	0.003
Triglycerides (mg/dL)	156 [118–211]	152 [120–196]	138 [98–151]	0.108
***CYP7A1***	***rs3808607 G/T***			
	*TT* **(*n* ═ *211*)**	****GT* (*n* ═ *167*)***	****GG* (*n* ═ *38*)***	P*
BMI (kg/m^2^)	28 [26–31]	28 [26–30]	28 [26–31]	0.358
Systolic blood pressure (mmHg)	121 [111–133]	121 [112–131]	127 [110–139]	0.208
Diastolic blood pressure (mmHg)	75 [69–81]	74 [67–80]	75 [69–85]	0.413
Glucose (mg/dL)	93 [87–108]	94 [86–103]	94 [86–104]	0.708
Total cholesterol (mg/dL)	193 [165–222]	202 [168–218]	203 [183–213]	0.593
HDL-cholesterol (mg/dL)	43 [36–50]	43 [36–50]	47 [39–54]	0.157
LDL-cholesterol (mg/dL)	120 [100–145]	129 [106–145]	143 [129–147]	0.006
Triglycerides (mg/dL)	159 [116–216]	157 [123–198]	134 [99–160]	0.044

Control subjects were not included in the subanalyses. Variables are expressed median [25th–75th interquartile interval] and logarithmically transformed for *ANOVA analysis. BMI: Body mass index; HDL: High-density lipoprotein; LDL: Low-density lipoprotein.

Since diabetes is a major risk factor for coronary artery disease that may bias the results, we performed a sub-analysis excluding these patients to confirm the contribution of genotypes in nondiabetic subjects (Tables S3 and S4). In this subgroup, the Hardy–Weinberg equilibrium was conserved and glucose plasma levels and HDL-C were no longer different between SA and control subjects (Table S3). Importantly, five out of the seven studied polymorphisms remained associated with SA under similar inheritance models compared to the whole group (Table S4). Consistently, the minor allele of any of the seven polymorphic sites was associated with higher LDL-C plasma levels in the SA patients after excluding patients with diabetes from the analysis.

## Discussion

In this study, we evaluated whether seven polymorphisms located in the promoter and enhancer regions of the *CYP7A1* gene were associated with plasma lipid levels and the incidence of SA. These SNPs encode cholesterol 7α-hydroxylase, a key enzyme in cholesterol catabolism, bile acid homeostasis, and plasma lipid levels [9–17]. The association of these *CYP7A1* polymorphisms with cardiovascular diseases has been mostly explained by increased LDL-C levels. In our study, we determined that the minor allele frequencies conferred an increased risk of developing SA. In addition, the association of these polymorphisms with SA in other populations has not been reported; our work is one of the few studies that describes the statistical relationship between these polymorphisms and coronary artery disease, acute coronary syndrome, hypercholesterolemia or diabetes [10, 12–17, 25]. Focusing on the rs2081687 *T* allele, which was associated with the risk of SA, several studies have shown the association of this allele with the risk of CAD and acute coronary syndrome [16, 25–28]. In addition, an experimental study showed that this same allele was associated with high LDL-C plasma concentrations [28]. Accordingly, we observed that patients with SA who were homozygous for the rs2081687 *T* allele, had higher plasma LDL-C plasma concentrations than in heterozygotes or non-carrier patients.

Concerning the rs8192870 polymorphism, recent studies revealed that the *T* allele was associated with an increased risk of diabetes, and acute coronary syndrome [17, 25]. In our study, the rs8192870 *T* allele homozygous patients also had elevated LDL-C plasma levels than heterozygotes or noncarriers of this allele. Regarding the rs10504255 *A/G* SNP, in our study, the *GG* genotype was statistically associated with a higher risk of SA, as well as with increased LDL-C levels. Similar results were recently reported by our group in patients with ACS [25]. Conversely, Wang et al. [10] reported that both, *G* and *A* alleles, did not differ in their gene expression regulation effects, as demonstrated by cloning each sequence into the reporter gene pGL4.23. In this context, we consider that the rs10504255 *A/G* SNP is physiologically relevant and merits being explored in other populations with different cardiovascular diseases.

The existing information about the relationship between the rs9297994 *G/A* and rs10107182 *C/T* polymorphisms with LDL-C plasma levels and cardiovascular diseases is scarce [10]. Genome-wide association studies revealed that both polymorphisms may be related to total cholesterol and LDL-C plasma levels, and an increased incidence of cardiovascular diseases [29–32]. Moreover, Wang et al. [10] reported that the rs9297994 *G/A* and rs10107182 *C/T* SNPs are in LD with the rs3808607 polymorphism, which is involved in the regulation of *CYP7A1* gene expression. Assuming that low *CYP7A1* mRNA expression results in higher levels of plasma cholesterol, our findings are congruent with these results: the rs9297994 *GG,* rs10107182 *CC*, and rs3808607 *GG* genotypes were associated with higher LDL-C levels in patients with SA. This association was not observed in patients with acute coronary syndrome [25] probably because of the use of anti-dyslipidemic and anti-hypertensive drugs, among others, in these subjects. Taken together, this evidence suggests that these polymorphisms can be useful to constitute a genetic panel for the evaluation of the risk of developing symptomatic disease in combination with other polymorphisms. This possibility remains to be explored in future studies.

Concerning the rs3808607 *G/T* SNP, previous studies have shown that the *G* allele is associated with higher plasma LDL-C plasma levels [10, 33] but its association with cardiovascular diseases or DM2 remains controversial [15, 16, 34]. In this context, our study showed that not only the homozygous carriers of the rs3808607 *G* allele had higher levels of LDL-C, but also the rs9297994 *GG* and rs10107182 *CC* genotypes were associated with increased LDL-C levels. Consistently, these same genotypes, i.e., rs3808607 GG, rs9297994 *GG*, and rs10107182 *CC*, were more frequent in patients with SA. This association may be due to the high LD that exists between these SNPs [10]. In line with this data, a recent case-control study demonstrated that rs9297994 and rs10107182 were associated with the risk of developing ACS [25].

In contrast with our results, a previous report did not find any association between the rs1457043 *C/T* polymorphism and the prevalence of coronary heart disease [10]. We observed that the *C* allele was associated with higher plasma LDL-C levels and with an increased risk of developing SA. We also determined that the “*TGCGCTG*” haplotype, formed by the seven studied polymorphisms, was associated with an increased risk of developing SA, and a high probably that this block could segregate together (D’ > 0.85). As far as we know, there are no studies that showed a haplotype similar to the one reported in our study. In this context, taken together, our results suggest a link between *CYP7A1* polymorphisms and the incidence of SA through plasma LDL-C levels. Nonetheless, previous studies have proposed that the possible mechanism is the effect of *CYP7A1* gene polymorphisms on *CYP7A1* mRNA expression, and plasma lipid concentrations [10, 11, 28–33].

When SA patients were grouped by genotype, we observed significantly higher levels of LDL-C in carriers of any of the seven studied alleles. Taken together, our results suggest that polymorphisms located in the enhancer/promoter region of the *CYP7A1* gene increase the risk of AS by favoring a slight but significant increase in plasma cholesterol levels. Since CYP7A1 polymorphisms have been associated with diabetes, and given that the SA and control groups were different in diabetes frequency [15, 25], we performed a statistical sub-analysis that excluded patients with diabetes. Even with lower statistical power, the minor alleles of any of the seven polymorphic sites were consistently associated with higher LDL-C plasma levels in the SA patients. These results further support the idea that polymorphic sites within the promoter/enhancer region of CYP7A1 contribute to SA via LDL-C plasma levels, independently of the incidence of diabetes.

On the other hand, the contribution of the studied polymorphisms to dyslipidemia and cardiovascular diseases remains controversial, likely due to ethnicity. In this context, the distribution of the minor alleles of the seven studied polymorphisms was lower in our population (Mexican mestizos) compared to Asian and Caucasian populations (Table S5). Nonetheless, in the African population, the distribution of the rs2081687 *T,* rs1457043 *C*, and rs3808607 *G* alleles was higher than in ours, but the rs9297994 *G*, rs10107182 *C,* and rs10504255 *G* alleles were much lower compared to other populations (Table S5). Taken together, our results and the different distribution of *CYP7A1* polymorphisms based on ethnicity, suggest that the effect of these SNPs on SA and other cardiovascular diseases merits further exploration in a multicentric study involving patients of diverse origins.

### Limitations of the study

We recognize that our study has some limitations that merit consideration: 1) As a consequence of the low frequency of the minor alleles in the studied population, there were few carriers of the genotypes and haplotypes associated with the risk of SA. 2) This study was not matched by sex and age; the proportion of men to women in the SA group was higher than in the control group. Even if these variables were considered to adjust the statistical analysis, this issue should be considered when interpreting of the study results. 3) This study did not demonstrate a cause–effect relationship between *CYP7A1* polymorphisms and LDL-C plasma levels and SA risk; instead, it only showed a statistical association between variables. Therefore, future experimental studies, such as GWAS, exome sequencing studies, and exome chips, are needed to ensure the validity, reliability, and accuracy of these polymorphisms in clinical practice, but also functional studies that demonstrate the effect of the polymorphisms on the *CYP7A1* mRNA expression.

## Conclusion

In conclusion, our results have shown that the rs2081687 *C/T,* rs9297994 *G/A*, rs10107182 *C/T,* rs10504255, *A/G* rs1457043 *C/T,* rs8192870 *G/T*, and rs3808607 *G/T* SNPs of the *CYP7A1* gene conferred an increased risk of developing SA, either individually or as part of a haplotype (*TGCGCTG*). In addition, our study showed the carrier individuals of the rs2081687 *TT,* rs9297994 *GG*, rs10107182 *CC,* rs10504255 *GG,* rs1457043 *CC,* rs8192870 *TT*, and rs3808607 *GG* genotypes had increased LDL-C levels in our population. Lastly, based on our results and the genetic distribution of these polymorphisms in our population, these SNPs deserve to be studied in populations of different ethnic origins to establish the right role of these polymorphisms in susceptibility to developing SA and other cardiovascular diseases.

## Supplemental data

Supplementary data is available at the following link: https://www.bjbms.org/ojs/index.php/bjbms/article/view/10764/3455

## Data Availability

Data of this study are accessible upon requirement to the author of correspondence.
